# Flow Cytometry as the Tool to Define Peripheral Blood Leukocyte Signatures in Acute EBV Infection

**DOI:** 10.3390/cells13110963

**Published:** 2024-06-03

**Authors:** Pragya Singh, Manisha Gadgeel, Batool AlQanber, Ahmad Farooqi, Süreyya Savaşan

**Affiliations:** 1 Children’s Hospital of Michigan, Detroit, MI 48201, USA; singhp13@chp.edu; 2 Hematology/Oncology Flow Cytometry Laboratory, Division of Hematology/Oncology, Children’s Hospital of Michigan, Detroit, MI 48201, USA; gadge1m@cmich.edu (M.G.); alqan1b@cmich.edu (B.A.); 3 Department of Pediatrics, Central Michigan University College of Medicine, Saginaw, MI 48602, USA; faroo2a@cmich.edu; 4 School of Medicine, Wayne State University, Detroit, MI 48202, USA; 5 Barbara Ann Karmanos Cancer Center, Division of Hematology/Oncology, Pediatric Blood and Marrow Transplant Program, Children’s Hospital of Michigan, Detroit, MI 48201, USA

**Keywords:** EBV, flow cytometry, T-large granular lymphocytes, monocytes, CD16, neutrophils, CD64

## Abstract

Primary Epstein–Barr virus (EBV) infection which can manifest as infectious mononucleosis (IM) is commonly acquired during childhood. EBV primarily invades B cells leading to a lytic reaction; the control of the infection is handled by natural killer and T cells in immunocompetent individuals. The infection has a wide spectrum of clinical findings and can lead to serious complications in patients with certain underlying immunological dysfunctions. We retrospectively investigated peripheral white blood cell populations’ surface marker characteristics in IM using a comprehensive flow cytometry marker panel. Twenty-one cases of IM and seventeen EBV-seropositive cases without IM serving as controls were included. We observed novel alterations in lymphocyte, neutrophil, and monocyte populations. In addition to increased activated cytotoxic T cells and low B cells, we demonstrated high T-large granular lymphocyte (T-LGL) populations in IM cases. Furthermore, despite T cells’ increased HLA-DR expression, another activation marker, CD11b, was lower in T-LGL populations. Monocytes showed increased CD16 expression; CD64 was higher in neutrophils. Our findings point to monocyte and neutrophil activation which may account for acute clinical features and may contribute to the understanding of IM immunobiology. Furthermore, they may serve as a useful tool in investigating inherited and post-transplant conditions characterized by deficiencies in controlling EBV infection.

## 1. Introduction

Epstein–Barr virus (EBV) is a B lymphotropic herpesvirus with a worldwide seroprevalence of above 90%. Primary EBV infection, which can manifest as infectious mononucleosis (IM), is usually acquired in childhood and stays in a latent form lifelong [1]. Most early childhood encounters are either asymptomatic or present as a self-resolving, acute upper respiratory infection. Adolescent infections are frequently characterized by the pathognomonic complex of a sore throat, fever, cervical lymphadenitis, fatigue, and hepatosplenomegaly. The virus initially replicates within the oropharyngeal epithelial cells and local B lymphocytes, followed by viremia through lytic replication. The host–virus balance is influenced by the immune status, age, and genetic susceptibility of the host.

While EBV infection provides both cellular and humoral immunity, it is the cellular response that results in the control of infection [2]. Polyclonal antibody production due to the EBV activation of B cells directed against antigens including viral capsid, nuclear, and early antigens is useful in the diagnosis and monitoring of infection in addition to polymerase chain reaction-based circulating EBV DNA detection. An exaggerated global CD8+ expansion is the hallmark of IM, the majority of which is directed towards EBV-specific antigens. There are conflicting data regarding peripheral NK cell (CD56-dim/CD16+) numbers in IM. However, their role in controlling infection is undisputed, as evidenced by the morbidity and outcomes of EBV disease in NK cell immunodeficiencies. Tonsillar NK cells (CD56-bright/CD16−) secrete IFN-γ, which retards the EBV-induced transformation of B cells and provides a first line of defense [3].

The above-mentioned cellular immunopathology of EBV infection has been documented in previous flow cytometry studies. However, with the advent of flow cytometric technology, a further detailed analysis of cellular immune response has become possible. In this study, we characterized cellular features of different white blood cell (WBC) types including but not limited to lymphocytes and established an EBV-specific predictive model based on flow cytometric characteristics using the most prominent alterations that occur during IM.

## 2. Materials and Methods

Institutional cases that had flow cytometric testing conducted for various clinical indications along with EBV serology evaluation, from January 2010 to June 2020, were reviewed retrospectively. Patients with an EBV serology test obtained within 30 days of flow studies were included in the analysis. IM and control cases were identified based on clinical presentation and serological test results. IM cases were characterized by the presence of EBV viral capsid antigen (VCA)-IgM positivity.

On the other hand, the control group consisted of EBV-seropositive cases without clinical evidence of acute infection, with negative EBV VCA-IgM and positive EBV VCA-IgG and EBV nuclear antigen (EBNA)-IgG. Flow cytometric list-mode reanalysis was performed in those cases. Cases with positive EBV VCA-IgM because of viral reactivation were not included in the analysis. Patients with underlying immune dysregulation were also excluded. The Human Investigation Committee at Wayne State University approval was obtained to review and report on cases which had flow cytometric analysis performed at Hematology/Oncology Flow Cytometry Laboratory at Children’s Hospital of Michigan.

A comprehensive flow cytometric panel of 33 surface markers was run to analyze any abnormality in peripheral blood lymphocytes, monocytes, and granulocytes in all the samples submitted to the laboratory for analysis. In this study, we report the surface marker expression patterns of CD45 (clone Immu19.2), CD3 (clone UCHT1), CD4 (clone 13B8.2), CD5 (clone BL1a), CD8 (clone B9.11), CD11b (clone Bear1), CD19 (clone J3-119), CD38 (clone T16), HLA-DR (clone B8.12), CD16 (clone 3G8), and CD56 (clone N901) in lymphocytes; CD45, CD13 (clone SJ1D1), and CD64 (clone 22) in granulocytes; and CD45, CD14 (clone RMO52), and CD16 in monocytes. All markers were purchased from Beckman Coulter; Brea, CA, USA. Briefly, mononuclear cells from heparinized peripheral blood samples were obtained by density gradient separation using Fico/Lite-LymphoH (Atlanta Biologicals; Flowery Branch, GA, USA) and suspended in complete medium (RPMI1640 +10% fetal bovine serum + gentamycin). Cell viability was assessed by trypan blue staining. Mononuclear cells were stained with above-mentioned fluorochrome-tagged monoclonal antibodies in different 10-color or 3-color combinations in the dark at room temperature for 20 min. Cells were then washed and resuspended in PBS and run on the flow cytometer.

**Figure 1:**
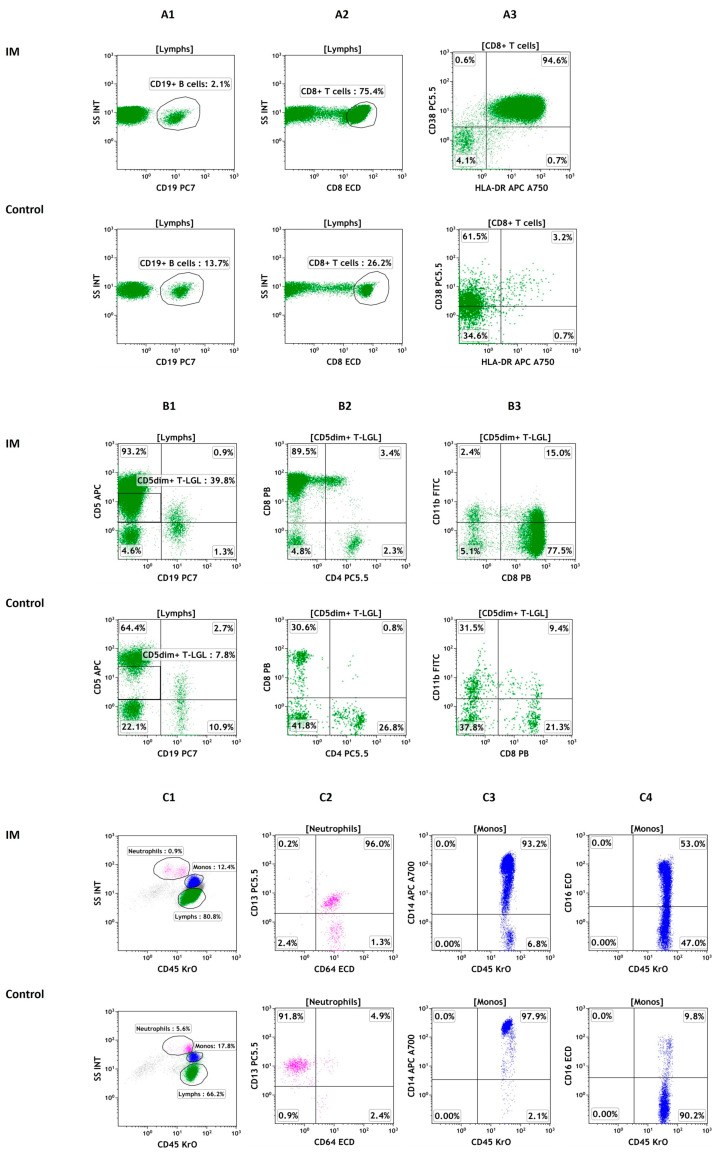
Flow cytometric analysis of lymphocytes, monocytes, and granulocytes in infectious mononucleosis and EBV-seropositive control patient. Flow cytometry dot-plots of B lymphocyte subsets in infectious mononucleosis and EBV-seropositive control patient with infectious mononucleosis patient showing decreased B cell content (**A1**). Flow cytometry dot-plots of CD8+ T lymphocyte subsets in infectious mononucleosis and EBV-seropositive control patient with infectious mononucleosis patient showing increased CD8 bright+ cytotoxic T cell content (**A2**). CD8 bright+ cytotoxic T cells displaying high HLA-DR/CD38 co-positive expression in infectious mononucleosis compared to seropositive control (**A3**). Flow cytometric characterization of CD5dim+ T-large granular lymphocytes (TLGL) in infectious mononucleosis and an EBV-seropositive patient. Infectious mononucleosis patient is showing increased CD5dim+ TLGL displaying high CD8+/CD11b- contents. Seropositive EBV patient is showing normal CD5dim+ TLGL content (**B1**–**B3**). Flow cytometry dot-plots show neutrophils gated on CD45/side scatter dot-plot and confirmed by positive CD13 staining. There is striking and almost uniform CD64 positivity in infectious mononucleosis case compared with very low expression in control case (**C1**,**C2**)). Flow cytometry dot-plots show monocytes gated on CD45/side scatter and confirmed by positive CD14 staining. While there is uniform high CD14 expression and lack of CD16 expression indicating presence of primarily classical monocytes in control case, there is sizeable cell population with positive CD16 expression on monocytes indicating presence of intermediate or non-classical monocytes in infectious mononucleosis (**C3**,**C4**)). Abbreviations: IM: infectious mononucleosis; Control: EBV-seropositive control; Lymphs: lymphocytes; Monos: monocytes.

Flow cytometry acquisition was performed on 3-color Beckman Coulter Epics XL flow cytometer in some cases and on 10-color Gallios flow cytometer (Beckman Coulter, Brea, CA, USA) in others due to the availability of the equipment. Both flow cytometers have given comparable results in repeated runs over several years of use in our laboratory. Evidently, 10-color Gallios flow cytometer provides higher analytic capability of samples run. Further detailed list-mode reanalysis of cases run on Gallios flow cytometer was conducted using Kaluza Analysis Software (version 1.3, Beckman Coulter, Brea, CA, USA). Lymphocyte populations were gated based on CD45/side scatter characteristics, followed by CD19/side scatter for CD19+ B cell reanalysis (Figure 1(A1)) and CD8/side scatter for CD8 bright+ cytotoxic T cell analysis. For cytotoxic T cell analysis, gating on CD3+ cells followed by CD8+ cells would provide more precise cytotoxic T cell gate as NK cells which are CD3- also express CD8. However, since this is a retrospective study, we had limitations with such gating strategy as CD3 and CD8 markers were not in the same tube. However, we observed that NK cells have dimmer CD8 expression compared to CD8 bright + cytotoxic T cells, and we restricted our gate around CD8 bright+ T cells only to overcome this limitation. CD8 bright+ T cells were further analyzed for HLA-DR/CD38 expression (Figure 1(A2,A3)). CD45bright+/CD19-neg/CD5dim+ T cells within lymphocyte gate, which represent T-Large granular lymphocytes (T-LGL), were analyzed for CD4+/CD8+ ratio and CD8/CD11b expression (Figure 1(B1–B3)). Monocytes and granulocytes were gated based on CD45/side scatter characteristics, and granulocytes were analyzed for surface expression of CD13 and CD64 (Figure 1(C1,C2)), while monocytes were analyzed for surface expression of CD14 and CD16 to assess classical, intermediate, and non-classical subsets. Because of retrospective nature of our study, we again had limitations describing these subsets precisely as CD14 and CD16 markers are not in same tube in our panel. We therefore restricted our description as CD16+ “intermediate” or “non-classical” monocytes to define these subsets. (Figure 1(C3,C4)). Positive surface marker expression was recorded in percentages.

SAS software (version 9.4, SAS Institute Inc., Cary, NC, USA) was used for data analysis, and significance level was set at 0.05. Data were summarized and reported with categorical variables by numbers and percentages. Normality of continuous variables was tested by Shapiro–Wilk test. Descriptive statistics for normally distributed continuous variables were reported with means and Standard Deviations, whereas non-normally distributed continuous variables were reported by Medians and Inter-Quartile Ranges. Two group comparisons for normal continuous variables were conducted using Student’s t-test, whereas non-normally distributed continuous variables were compared using Wilcoxon rank sum test. For multivariate analysis, logistic regression with odds ratio was used to determine effect sizes with 95% confidence interval. Student’s t test (2-tailed) and Pearson’s correlation were performed for assessment of statistical significance in cases with 10-color flow cytometry analysis.

## 3. Results

A total of 406 cases with available flow cytometric test results on peripheral blood samples were reviewed in this retrospective study. Based on clinical presentation and serological test results, 21 cases of IM and 17 EBV-seropositive cases without IM serving as controls were included in this study. In the IM group, flow cytometry study indications included suspected IM in 39%, lymphadenopathy in 28%, thrombocytopenia in 16%, the observation of immature cell morphology on peripheral blood in 11%, and leukocytosis in 6% cases. In control cases, 41% had thrombocytopenia, 24% lymphadenopathy, 17% the observation of immature cell morphology on peripheral blood, 12% a fever of unknown origin, and 6% leukocytosis as indications for flow cytometric analysis per the treating physician. Out of total 38 cases, flow cytometry acquisition was performed on a 3-color Beckman Coulter Epics XL flow cytometer in 22 cases and the remaining 16 cases (11 IM and 5 control cases) on a 10-color Gallios flow cytometer.

Among the relevant flow cytometric parameters compared between the IM and control group, eight showed significant difference. While lymphocyte CD3, CD8, HLA-DR expression, and HLA-DR/CD38 co-expression, CD5dim+ T-LGL content, monocyte CD16, and granulocyte CD64 expression was higher in the IM group along with the reversed CD4/CD8 ratio, lymphocyte CD4 and CD19 expression was higher in the control group. The CD4/CD8 ratio was calculated based on the %CD4 and %CD8 populations in CD4 vs. CD8 dot-plots. There was no difference in age and activated natural killer lymphocytes (CD56+CD16+) between the groups. Some of the values are summarized in Table 1.

The mean CD8+ T cell HLA-DR+CD38+ expression was much higher in IM than control cases in 10-color flow cytometry analysis (72.2 ± 25 vs. 15.6 ± 21; *p* = 0.001), indicating the significant activation status of cytotoxic T cells in IM. However, despite the predominance of CD8+ cells in the T-LGL population (81.5 ± 10 vs. 51.7 ± 16.9; *p* = 0.001), the great majority of T-LGL did not express another activation marker, CD11b (CD8+CD11b– T-LGL at 71 ± 14.7 in IM vs. 28.5 ± 8.8 in the controls; *p* = 0.001). The contrasting expression of activation markers HLA-DR and CD11b in IM is quite interesting (Figure 1(A3,B3)).

There was a significant increase in CD64-expressing neutrophils in IM cases compared with control cases (64.4% vs. 14.4%). Furthermore, in the IM group, a prominent CD16-expressing monocyte population was observed (25.8% vs. 14.7%; *p* = 0.0074) (Table 1) representing “intermediate” or “non-classical” monocytes (Figure 1(C4)).

**Figure 2:**
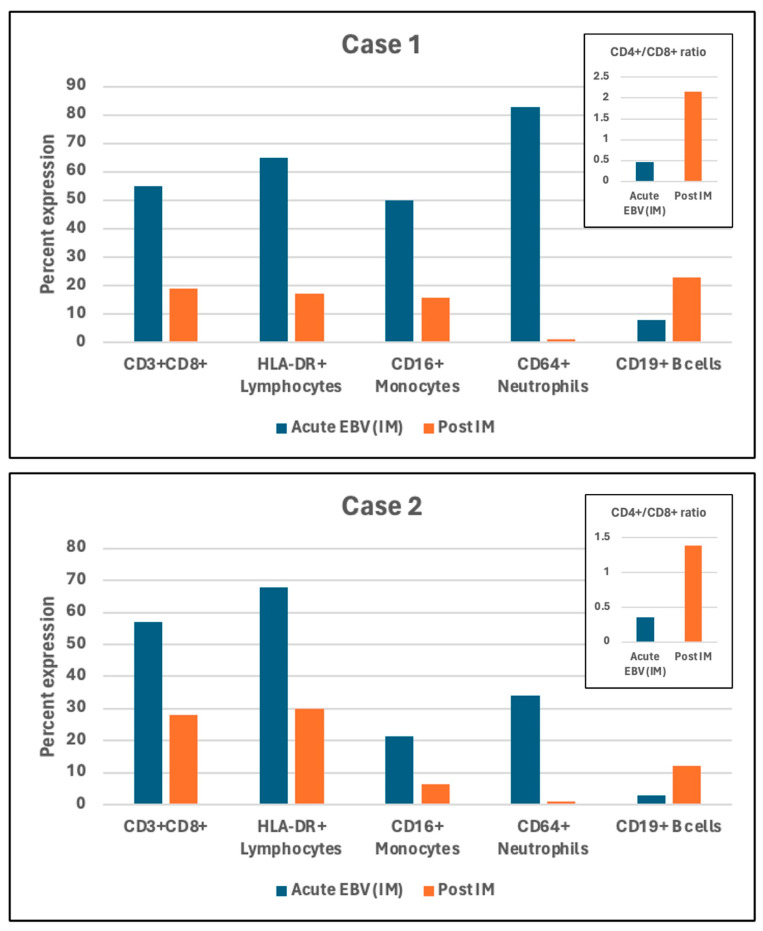
Changes in flow cytometric characteristics in two cases in one month after initial acute EBV infection diagnosis. Both Case 1 and Case 2 show decrease in cytotoxic activated CD3+CD8+ T cells, decreased HLA-DR+ activated lymphocytes, decreased CD16+ intermediate or non-classical monocytes, and striking disappearance of CD64+ neutrophils, increase in B cells as displayed in the bar graph one month post IM compared to that during active phase of EBV infection. Bar graph displays normalization of CD4/CD8 ratio one month post IM.

In two cases, repeat flow studies were obtained a month after the initial testing when acute EBV infection was established. The results showed the normalization of the changes observed during the acute phase characterized by an increase in B cells, decrease in cytotoxic activated T cells, and CD16+ intermediate or non-classical monocytes and a striking disappearance of CD64+ neutrophils (Figure 2).

A multiple logistic regression was performed on EBV status as an outcome variable to ascertain the effects of four predictors, which were noted to be significant in the univariate analysis: lymphocyte CD4+/CD8+ ratio, CD19 expression, T-LGL population size in the T cell gate, and granulocyte CD64 expression. According to the Wald criterion, only the CD19+ lymphocyte population size reliably predicted IM, keeping other predictors constant (OR = 0.729; CI = 0.537–0.991; *p* = 0.044).

## 4. Discussion

Infectious mononucleosis is essentially an acute and unique manifestation of the body’s immune response to primary EBV infection. The symptomatology of IM is quite variable given the intricate interplay between the host and the virus. However, certain cellular responses in IM appear to be consistent across multiple studies. These include the hallmark amplification of the main effector/cytotoxic T cells (CD3+CD8+). Not surprisingly, this was also significant in our study. This pattern was further confirmed by a low CD4+/CD8+ ratio of 0.5 compared to the control group ratio of 1.7.

Consistent with B lymphotropism and the lytic effect of EBV, B cells were significantly lower in the IM group. Furthermore, cytotoxic T cells expressed significantly higher CD38/HLA-DR double-positive expression in IM compared with control cases, indicating the activation status. However, another activation marker, CD11b, was not expressed highly on T-LGL in IM patients, as once mentioned by Ebihara et al. in T cells several years ago [4]. The differential expression of activation markers of HLA-DR and CD11b in IM is of interest and warrants further investigation. Furthermore, T-LGL was significantly higher in IM compared to control cases, 23.7% vs. 10.7% (*p* = 0.0004). We previously showed the clonal T-LGL expansion in cases with underlying immune deficiency/dysregulation/autoimmunity/lymphoproliferation [5].

Of note, there was a striking increase in CD64-expressing neutrophils in the IM group. CD64 is the Fc-gamma receptor-I and is involved in neutrophil activation. Increased neutrophil CD64 expression was found to be a reliable marker of sepsis in both neonates and adult patients admitted in intensive care units [6,7]. Neutrophil CD64 expression is associated with bacterial infections; recent evidence suggests there is a relationship with non-infectious inflammatory conditions as well [8,9]. Thus, our finding of increased neutrophil CD64 expression and its rapid normalization within a one-month period may be another component of the inflammatory process and associated clinical symptoms in IM. Nevertheless, it is concluded that neutrophil CD64 expression cannot be used to distinguish between bacterial and viral infections [10].

Additionally, a marked increase was noted in the frequency of CD16+ intermediate or non-classical monocytes in IM cases. CD16 is an Fc-gamma receptor-III and an activation marker for monocytes [11]. Intermediate monocytes are known to have pro-inflammatory characteristics, having antigen presentation capacity, which may be an important role that they play in IM. Since almost all intermediate monocytes progress into non-classical monocytes which possess anti-viral activities along with migration into tissues, they may bear an important contribution to IM pathology [12].

A multiple logistic regression analysis using the lymphocyte CD4+/CD8+ ratio, CD19 expression, T-LGL population size in the T cell gate, and granulocyte CD64 expression was performed on EBV status as they were noted to be significant variables between IM and control cases in the univariate analysis. Only the CD19+ lymphocyte population, B cell content reliably predicted the diagnosis of IM cases keeping other predictors constant. Though serological testing and EBV DNA quantification are reliable diagnostic tools, in certain cases, especially when a lymphoproliferative process is suspected, the flow cytometric profile from our study of EBV-associated changes may help in directing towards EBV testing. Including flow cytometric LMP1 staining in B, cytotoxic T, and T-LGL populations during the acute and late/recovery phases of the infection may shed light on the potential course of EBV infection particularly in patients with underlying immune dysregulation.

## 5. Conclusions

In summary, acute EBV infection is characterized by increased activated cytotoxic T cells with a differential expression of certain activation markers, HLA-DR and CD11b, decreased B cells, expanded T-LGL, and activated monocytes and granulocytes, which may account for several immunologic and inflammatory reflections in the clinical picture. Among these, the expansion of T-LGL is of interest since there is evidence of their potential role in controlling the pathological process. The findings of this study expand the cellular activation profile in acute EBV infection beyond cytotoxic T cells into monocytes and granulocytes. The unique flow cytometric pattern seen here may also be a useful tool in the research of immune dysfunction conditions associated with uncontrolled EBV infection.

## Figures and Tables

**Table 1:** Comparisons between infectious mononucleosis and seropositive control group variables.

	IM (N = 21)	Control (N = 17)	*p* Value
Age (years)	9.3 (1.3–17.8)	10.5 (1.8–21.2)	*p* > 0.05
Lymphocyte gate CD3+	83% (68–94)	66.6% (50–80)	*p* < 0.0001
Lymphocyte gate CD8+	55.5% (32–80)	27.6% (12–42)	*p* < 0.0001
Lymphocyte gate CD4+/CD8+ ratio	0.5 (0.1–1.5)	1.7 (0.8–3.8)	*p* < 0.0001
Lymphocyte gate HLA-DR+	55.6% (19–87)	21.6% (13–52)	*p* < 0.0001
Lymphocyte gate CD19+ (B cells)	6% (1–14)	18.8% (6–32)	*p* < 0.0001
Lymphocyte gate CD56+CD16+ (NK cells)	7.4% (2–17)	9.9% (0.1–28)	*p* > 0.05
Lymphocyte gate HLA-DR+CD38+	54 (17–84.1)	16.8% (7.9–46.3)	*p* < 0.0001
T cell gate T-LGL	23.7% (7.5–54)	10.7% (4.8–23.5)	*p* = 0.0004
Monocyte gate CD16+	25.8% (7.1–54.2)	14.7% (5.3–37.6)	*p* = 0.0074
Neutrophil gate CD64+	64.2% (6–95.1)	14.4 (0.4–80.8)	*p* < 0.0001

Abbreviations: IM: infectious mononucleosis; T-LGL: T-large granular lymphocytes. Numbers in the upper panel represent mean values with ranges.

## Data Availability

The raw data supporting the conclusions of this article will be made available by the authors on request.
